# Isothiocyanates Reduce Mercury Accumulation via an Nrf2-Dependent Mechanism during Exposure of Mice to Methylmercury

**DOI:** 10.1289/ehp.1003123

**Published:** 2011-03-07

**Authors:** Takashi Toyama, Yasuhiro Shinkai, Akira Yasutake, Koji Uchida, Masayuki Yamamoto, Yoshito Kumagai

**Affiliations:** 1 Doctoral Programs in Medical Sciences, Graduate School of Comprehensive Human Sciences, University of Tsukuba, Tsukuba, Japan; 2 Research Fellow of the Japan Society for the Promotion of Science, Tokyo, Japan; 3 Department of Basic Medical Sciences, Biochemistry Section, National Institute for Minamata Disease, Minamata, Japan; 4 Graduate School of Bioagricultural Sciences, Nagoya University, Aichi, Japan; 5 Department of Medical Biochemistry, Tohoku University Graduate School of Medicine, Sendai, Japan

**Keywords:** chemoprevention, glutathione, methylmercury, Nrf2, 6-methylsulfinylhexyl isothiocyanate, sulforaphane

## Abstract

Background: Methylmercury (MeHg) exhibits neurotoxicity through accumulation in the brain. The transcription factor Nrf2 (nuclear factor erythroid 2-related factor 2) plays an important role in reducing the cellular accumulation of MeHg.

Objectives: We investigated the protective effect of isothiocyanates, which are known to activate Nrf2, on the accumulation of mercury after exposure to MeHg *in vitro* and *in vivo*.

Methods: We used primary mouse hepatocytes in *in vitro* experiments and mice as an *in vivo* model. We used Western blotting, luciferase assays, atomic absorption spectrometry assays, and MTT [3-(4,5-dimethylthiazol-2-yl)-2,5-diphenyl tetrazolium bromide] assays, and we identified toxicity in mice based on hind-limb flaccidity and mortality.

Results: The isothiocyanates 6-methylsulfinylhexyl isothiocyanate (6-HITC) and sulforaphane (SFN) activated Nrf2 and up-regulated downstream proteins associated with MeHg excretion, such as glutamate-cysteine ligase, glutathione *S*-transferase, and multidrug resistance–associated protein, in primary mouse hepatocytes. Under these conditions, intracellular glutathione levels increased in wild-type but not Nrf2-deficient primary mouse hepatocytes. Pretreatment with 6-HITC and SFN before MeHg exposure suppressed cellular accumulation of mercury and cytotoxicity in wild-type but not Nrf2-deficient primary mouse hepatocytes. In comparison, *in vivo* administration of MeHg to Nrf2-deficient mice resulted in increased sensitivity to mercury concomitant with an increase in mercury accumulation in the brain and liver. Injection of SFN before administration of MeHg resulted in a decrease in mercury accumulation in the brain and liver of wild-type, but not Nrf2-deficient, mice.

Conclusions: Through activation of Nrf2, 6-HITC and SFN can suppress mercury accumulation and intoxication caused by MeHg intake.

Monomethylmercury (MeHg) is a potent neurotoxicant that is rapidly taken up by organisms living in aquatic environments and is biomagnified through the food chain, reaching concentrations in some fish 10,000–100,000 times greater than in the surrounding water (Scheuhammer et al. 2007). In humans, exposure to high concentrations of MeHg causes central nervous system dysfunction because MeHg readily crosses the blood–brain barrier via the L-type large neutral amino acid transporter and accumulates in the brain (Simmons-Willis et al. 2002). In the past there have been accidental MeHg poisonings such those in Minamata, Japan, (Harada 1978) and Iraq (Bakir et al. 1973); however, recent concerns focus on the risk to human health from the accumulation of MeHg through daily consumption of large predatory fish such as tuna and swordfish (Grandjean et al. 2010). The mechanism of MeHg toxicity is, in part, thought to involve the covalent interaction of MeHg with the reactive thiols of certain proteins (Kanda et al. 2008; Shinyashiki et al. 1996; Vogel et al. 1985). Unbound MeHg undergoes conjugation with glutathione (GSH), which is synthesized by glutamate-cysteine ligase (GCL) to form a polar MeHg–GSH adduct (Rabenstein and Fairhurst 1975) through nonenzymatic processes and possibly enzymatic processes involving GSH *S*-transferases (GSTs). The MeHg–GSH adduct is thought to be excreted into the extracellular space via multidrug resistance–associated proteins (MRPs) (Ballatori 2002; Madejczyk et al. 2007; Vollrath et al. 2006).

The current consensus is that GCL (the rate-limiting enzyme for GSH synthesis), phase II detoxification enzymes such as GSTs, and phase III MRP transporters are coordinately regulated by the transcription factor Nrf2 (nuclear factor erythroid 2-related factor 2] (Itoh et al. 1997, 2004; Maher et al. 2007). Under basal conditions, Nrf2 is bound to Kelch-like ECH-associated protein 1 (Keap1), the negative regulator of Nrf2, and it undergoes degradation by the ubiquitin/proteasome system in the cytoplasm (Itoh et al. 2004). When the reactive thiol groups of Keap1 are modified by electrophiles and/or reactive oxygen species, Nrf2 is readily translocated into the nucleus, where it stimulates the antioxidant-responsive element (ARE) in the promoter region. We previously reported that activation of Nrf2 may be a key factor in detoxification of MeHg because Nrf2 facilitates the excretion of MeHg into the extracellular space in human neuroblastoma SH-SY5Y cells and primary mouse hepatocytes (Toyama et al. 2007). This observation has been further confirmed in primary rat astrocytes and microglial cells (Ni et al. 2010; Wang et al. 2009).

Evidence indicates that the activation of Nrf2 by chemopreventive agents is effective against various stresses and diseases (Kensler et al. 2007), and studies have shown that Nrf2 activators are capable of protecting against carcinogenesis in an Nrf2-dependent manner (Kwak and Kensler 2010; Ramos-Gomez et al. 2001). Isothiocyanates (ITCs) are among the most potent Nrf2 activators. For example, sulforaphane (SFN), an ITC found in broccoli sprouts, activates Nrf2 and up-regulates detoxifying enzymes, resulting in the reduction of arsenic accumulation and cytotoxicity in primary mouse hepatocytes (Shinkai et al. 2006). We hypothesize, therefore, that ITC-mediated activation of Nrf2 and up-regulation of the genes downstream of Nrf2 reduce cellular and organ mercury levels after exposure to MeHg, thereby diminishing the toxicity of this substance. We report here that SFN and 6-methylsulfinylhexyl isothiocyanate (6-HITC), an analogue of SFN isolated from wasabi (Japanese horseradish) that is also a potent Nrf2 inducer (Morimitsu et al. 2002), effectively suppress mercury accumulation after exposure to MeHg both *in vitro* and *in vivo*, leading to decreased cytotoxicity and intoxication through Nrf2 activation.

## Materials and Methods

*Materials.* MeHg was purchased from Nacalai Tesque (Kyoto, Japan), and SFN was obtained from LKT Laboratories (St. Paul, MN, USA). We purchased anti-GCL modifier subunit (GCLM), anti-GCL catalytic subunit (GCLC), anti-MRP2, and anti–5´-nucleotidase (5´NT) from Santa Cruz Biotechnology (Santa Cruz, CA, USA). We obtained anti-MRP1 from Alexis Biochemicals (San Diego, CA, USA) and anti-actin from Sigma (St. Louis, MO, USA). Anti-GSTA1 was purchased from Oxford Biomedical Research (Oxford, MI, USA). 6-HITC was prepared as described by Shibata et al. (2008). All other reagents and chemicals used were of the highest grade available.

*Cells and cell culture.* Primary hepatocytes were isolated from 6- to 10-week-old C57BL/6J male mice by two-step collagenase perfusion as described by Shinkai et al. (2009). Parenchymal hepatocytes were separated from nonparenchymal cells by differential centrifugation 50 × *g* for 3 min. Dead parenchymal hepatocytes were removed by density gradient centrifugation in Percoll. Final preparations were suspended at 4.0 × 10^5^ cells/mL in Williams medium E supplemented with 10% fetal bovine serum, 2 mM l-alanyl- l-glutamine, and antibiotics (100 U/mL penicillin, 100 μg/mL streptomycin) and then seeded at a density of 8 × 10^4^ cells/cm^2^ on a culture plate. Cultured cells were maintained at 37°C in a humidified incubator under an atmosphere of 5% CO_2_/95% ambient air. Before MeHg exposure, the cells were cultured in serum-free medium overnight and then exposed to buthionine sulfoximine (BSO), ethacrynic acid (EA), MK-571, SFN, or 6-HITC in serum-free medium.

*Measurement of mercury concentration.* Cells were washed twice with phosphate-buffered saline (PBS) and solubilized in 1 mL NaOH (sodium hydroxide; 0.3 N). Mouse organs were solubilized with 0.5 mL NaOH (2 N). An aliquot of the solution was used in mercury measurement by the oxygen combustion–gold amalgamation method using an atomic absorption mercury detector (model MD-A; Nippon Instruments, Osaka, Japan), and adjustments were made for protein concentrations as described by Fujiyama et al. (1994). Protein concentration was determined as described by Lowry et al. (1951), with bovine serum albumin as the external standard.

*Cell viability.* We used the MTT [3-(4,5-dimethylthiazol-2-yl)-2,5-diphenyl tetrazolium bromide) assay to estimate cell viability, as described by Shinkai et al. (2009).

*Western blot analysis.* After treatment, cells were washed twice with ice-cold PBS and solubilized with sodium dodecyl sulfate (SDS) sample buffer [50 mM Tris-HCl (pH 6.8), 2% SDS, 10% glycerol] to obtain total cellular protein. A crude membrane fraction was prepared by differential centrifugation, as described by Shinkai et al. (2009). Briefly, cells were scraped into PBS, resuspended in hypotonic lysis buffer [10 mM Tris-HCl (pH 7.5), 10 mM NaCl, 1 mM MgCl_2_], and incubated on ice for 15 min. Swollen cells were ruptured with 20 strokes in a tightly fitting Dounce homogenizer, and the nuclei were removed by centrifugation at 400 × *g* for 10 min at 4°C. The pellet obtained by subsequent centrifugation at 30,000 × *g* for 30 min at 4°C was used as the crude membrane fraction. Protein concentration was determined using bicinchoninic acid protein assay reagent (Pierce, Rockford, IL, USA) with bovine serum albumin as the standard. Proteins were separated by SDS/PAGE. The blots were blocked for 1 hr with 5% skim milk in Tween-Tris–buffered saline [TTBS; 20 mM Tris (pH 8.0), 150 mM NaCl, 0.5% Tween 20]. Blots were incubated with the indicated primary antibodies, washed with TTBS, and incubated with horseradish peroxidase–conjugated secondary antibody. Immunoreactive bands were visualized by enhanced chemiluminescence (Chemi-Lumi One; Nacalai Tesque) and scanned using an LAS-4000 imaging system (Fujifilm, Tokyo, Japan). The bands were quantified by using ImageJ software, version 1.37 (Rasband 2010), and the density of each band was normalized to that of actin. Representative blots are shown from three independent experiments.

*Luciferase assay.* We performed DNA transfections using Lipofectamine 2000 (Invitrogen, Carlsbad, CA, USA) according to the manufacturer’s instructions. Briefly, cells were cultured in 12-well plates. Two micrograms of ARE-luciferase cDNA and 0.2 μg pRL-TK cDNA or 4 μL transfection reagent were mixed with serum-free media. Before addition to the cells, the DNA solution and transfection reagent solution were mixed together and incubated for 20 min at room temperature to allow the formation of complexes. The complexes were then mixed with the culture media and incubated for 24 hr to transfect. After transfection, the cells were treated with 6-HITC or SFN for 12 hr, and then luciferase activity was measured in cellular extracts (Dual-Luciferase reporter assay system; Promega, Madison, WI, USA).

*Measurement of intracellular GSH.* Intracellular GSH content was measured as described by Vignaud et al. (2004), with slight modification. Briefly, we used an HPLC system (Simadzu, Kyoto, Japan) linked to a coulometric detector (Coulochem II; ESA, Chelmsford, MA, USA). Cells were washed twice with PBS and collected in 1 mM EDTA. After sonication, protein concentrations were measured by bicinchoninic acid (BCA) protein assay. Cell lysates were filtrated by using an Ultrafree-MC 5,000-MW filter unit (Millipore, Billerica, MA, USA) and then mixed with the mobile phase to 50%. Ten-microliter samples were loaded onto a C18 YMC HPLC column (250 mm × 4.6 mm, i.d.; YMC, Kyoto, Japan) equipped with a guard column (17 mm × 4.6 mm i.d.). Elution was performed isocratically, with 98% of the mobile phase containing 20 mM ammonium phosphate solution adjusted to pH 2.5 with orthophosphoric acid, and the remaining 2% of the mobile phase containing acetonitrile. The flow rate was fixed at 0.6 mL/min.

*Animals.* We used 6- to 10-week-old male homozygous (–/–) Nrf2-deficient mice (C57BL/6J) and wild-type (+/+) mice. DNA was taken from the tail of each mouse and analyzed by polymerase chain reaction to confirm genotype. The mice were housed in plastic cages in a climate-controlled animal room (temperature, 24°C ± 1°C; humidity, 55% ± 5%) with a 12-hr light/dark cycle (lights on at 0700 hours and off at 1900 hours). Food (Certified diet M; Oriental Yeast, Tokyo, Japan) and water were made freely available to the mice. MeHg dissolved in PBS was administered via oral intubation; SFN dissolved in corn oil was intraperitoneally injected into the mice. All animal protocols were approved by the University of Tsukuba Animal Care and Use Committee and performed with strict adherence to its guidelines for alleviation of suffering.

*Statistical analysis.* Statistical significance was assessed by Student’s *t*-test or chi-square test, and *p* < 0.05 was considered statistically significant.

## Results

**Figure 1:**
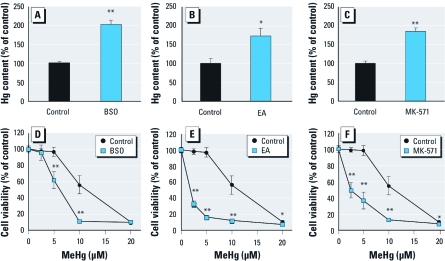
Effects of BSO (*A,D*), EA (*B,E*), and MK-571 (*C,F*) on mercury accumulation and cell viability in primary mouse hepatocytes after exposure to MeHg. (*A*–*C*) Cells were pretreated with BSO (500 μM) for 12 hr (*A*), EA (150 μM) for 1 hr (*B*), or MK-571 (100 μM) for 1 hr (*C*) before exposure to MeHg (10 μM) for 1 hr. The cells were further incubated without MeHg for 1 hr to enhance the efflux of mercury; then cellular accumulation of mercury was measured. (*D*–*F*) Cells were treated with BSO (500 μM) for 12 hr (*D*), EA (150 μM) for 1 hr (*E*), or MK-571 (100 μM) for 1 hr (*F*) before exposure to MeHg (2.5, 5, 10, or 20 μM) for 24 hr. An MTT assay was then performed. Each value is the mean ± SE of three determinations. *p < 0.05 and **p < 0.01 compared with control.

*Effects of inhibitors on MeHg accumulation and toxicity.* The major detoxification pathway for MeHg is conjugation with GSH derived from GCL in the presence or absence of GSTs, followed by excretion of the MeHg–GSH adduct into the extracellular space via MRPs. To confirm this, we first examined whether inhibition of GSH production, or GST or MRP activity, affects intracellular levels of MeHg (evaluated by determination of total mercury content) and MeHg-induced cytotoxicity *in vitro*. Pretreatment with BSO (a specific GCL inhibitor), EA (a GST inhibitor), or MK-571 (an MRP antagonist) resulted in significant enhancement of mercury accumulation in primary mouse hepatocytes exposed to MeHg (Figure 1A–C). Under these conditions, the cells were significantly more sensitive to MeHg, and cell viability decreased (Figure 1D–F). These results indicate that GCL, GST, and MRP contribute to cellular protection against MeHg toxicity through the reduction of mercury accumulation.

**Figure 2:**
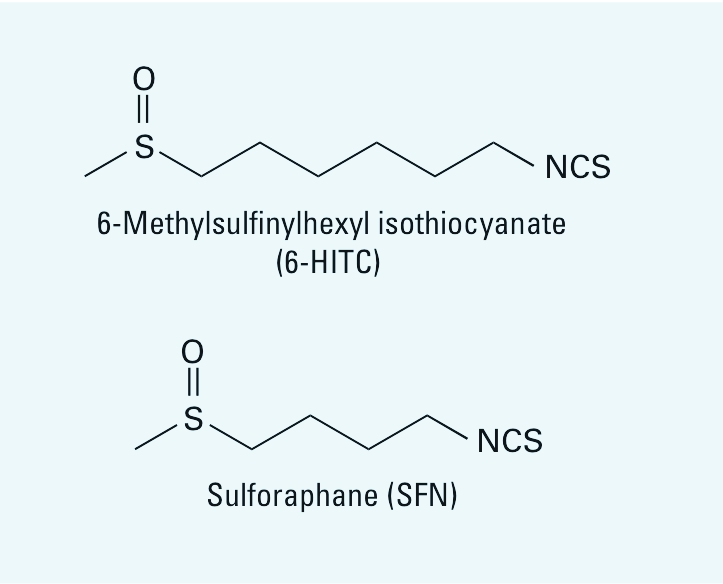
Chemical structures of 6‑HITC and SFN.

**Figure 3:**
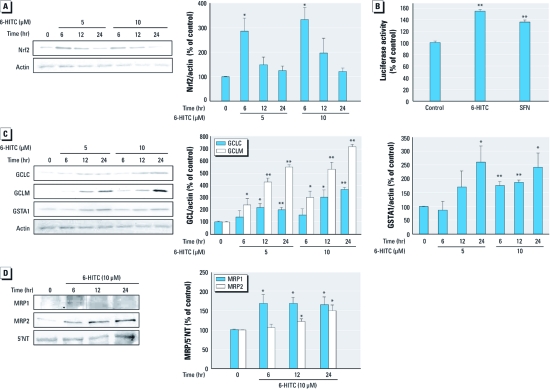
Effect of the ITCs 6‑HITC and SFN in primary mouse hepatocytes. (*A*) Total cell lysates from cells incubated with 6‑HITC (5 or 10 μM) for 6, 12, or 24 hr were subjected to Western blot analysis with anti-Nrf2. (*B*) Luciferase activity in primary mouse hepatocytes transfected with ARE-luciferase and pRL-TK cDNA and treated with ITCs (10 μM) for 12 hr. (*C*) Total cell lysates from cells incubated with 6‑HITC (5 or 10 μM) for 6, 12, or 24 hr were subjected to Western blot analysis using antibodies to GCLC, GCLM, and GSTA1. For *A* and *C*, anti‑actin was used as the internal control. (*D*) Crude membrane fractions of cells incubated with 6‑HITC (10 μM) for 6, 12, or 24 hr were subjected to Western blot analysis using the antibodies to MRP1 and MRP2; anti–5´NT was used as the internal control. Values shown are mean ± SE of three determinations. *p < 0.05, and **p < 0.01 compared with control.

*ITCs activate Nrf2 in primary mouse hepatocytes.* Because GCL, GSTs, and MRPs are regulated by Nrf2, we investigated the effects of the ITCs 6-HITC and SFN (Figure 2) on activation of Nrf2 and up-regulation of GCL, GSTs, and MRPs (Figure 3). Exposure of primary mouse hepatocytes to 6-HITC for 6 hr resulted in a significant increase in Nrf2 accumulation. The extent of the increase in Nrf2 accumulation was less at 12 and 24 hr than at 6 hr (Figure 3A). ARE luciferase activity was enhanced by exposure to 6-HITC or SFN for 12 hr (Figure 3B). Under these conditions, 6-HITC significantly increased the expression of GCLM, GCLC, and GSTA1 (Figure 3C) and MRP1 and MRP2 (Figure 3D) in primary mouse hepatocytes.

**Figure 4:**
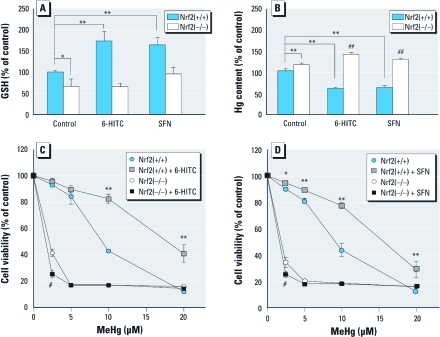
Nrf2-dependent effect of 12‑hr pretreatment with ITCs [6‑HITC (10 μM) or SFN (10 μM)] in primary hepatocytes from wild-type [Nrf2(+/+)] or Nrf2-deficient [Nrf2(–/–)] mice. (*A*) Cellular GSH concentration in cells incubated with ITCs. (*B*) Cellular accumulation of mercury in cells treated with ITCs and exposed to MeHg (10 μM) for 1 hr; cells were further incubated without MeHg for another 1 hr to enhance the efflux of mercury. (*C*) Cytotoxicity of MeHg in cells incubated with 6‑HITC and then exposed to MeHg (2.5, 5, 10, or 20 μM) for 24 hr and evaluated by MTT assay. (*D*) Cytotoxicity of MeHg in cells incubated with SFN and then exposed to MeHg (2.5, 5, 10, or 20 μM) for 24 hr and evaluated by MTT assay. Values shown are mean ± SE of three determinations. *p < 0.05, and **p < 0.01 compared with Nrf2(+/+) control. ^#^*p* < 0.05, and ^##^*p* < 0.01 compared with Nrf2(–/–) control.

*ITCs inhibit MeHg-induced mercury accumulation and cytotoxicity.* Because GCL is a rate-limiting enzyme for GSH synthesis, we measured intracellular GSH levels after exposure to ITCs in primary hepatocytes from wild-type and Nrf2-deficient mice. Both 6-HITC and SFN significantly increased the intracellular GSH level in wild-type cells, whereas GSH levels were not changed by ITCs in Nrf2-deficient cells (Figure 4A). This suggests that ITCs facilitate MeHg–GSH adduct formation and excretion into the extracellular space via an Nrf2-dependent pathway. To explore this possibility, we examined the effect of ITCs on mercury accumulation and cytotoxicity induced by MeHg exposure in primary hepatocytes from wild-type and Nrf2-deficient mice. Pretreatment with 6-HITC or SFN before exposure to MeHg resulted in a significant decrease in mercury accumulation (Figure 4B) and cytotoxicity (Figure 4C,D) in the wild-type cells, whereas no such protective effect was observed in Nrf2-deficient cells. To measure mercury accumulation, we exposed 6-HITC–pretreated or SFN-pretreated cells to MeHg for 1 hr and then incubated them in MeHg-free medium for an additional 1 hr. 6-HITC and SFN increased mercury accumulation and MeHg-induced cytotoxicity in the Nrf2-deficient cells. Because the Nrf2-deficient cells expressed lower basal levels of GCL, GST, and MRP than did the wild-type cells (data not shown), the basal level of intracellular GSH was also lower than that in the wild-type cells (Figure 4A). In nonpretreated primary hepatocytes from wild-type mice, mercury accumulation and cytotoxicity were increased by Nrf2 deficiency after MeHg exposure (Figure 4B,C).

**Figure 5:**
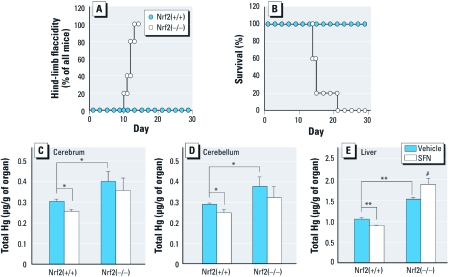
Effect of Nrf2 on MeHg intoxication and suppression of mercury accumulation by ITCs *in vivo*. (*A* and *B*) Hind-limb flaccidity (*A*) and mortality (*B*) in wild-type [Nrf2(+/+); *n* = 5] or Nrf2-deficient [Nrf2(–/–); *n* = 5] mice administered MeHg (5 mg/kg/day) for 8 days. (*C*–*E*) Mercury accumulation in the cerebrum (*C*), cerebellum (*D*), and liver (*E*) 48 hr after MHg exposure. For measurement of mercury accumulation, mice received a single injection of SFN (5 mg/kg) 16 hr before a single administration of MeHg (1 mg/kg). Values shown are mean ± SE of five determinations. *p < 0.05, and **p < 0.01 compared with Nrf2(+/+) control; ^#^*p* < 0.05 compared with Nrf2(–/–) control.

*Nrf2 suppresses MeHg intoxication* in vivo. To examine the contribution of Nrf2 to protection against MeHg toxicity *in vivo*, we counted the number of mice with flaccid hind limbs each day; this flaccidity is a typical sign of MeHg intoxication in rodents (Su et al. 1998). Oral administration of MeHg (5 mg/kg/day for 8 days) to Nrf2-deficient mice resulted in the induction of hind-limb flaccidity (Figure 5A), whereas wild-type mice did not show any abnormalities within 22 days after termination of MeHg treatment. The body weight of Nrf2-deficient mice 5 days after MeHg administration was approximately 30% less than that of wild-type mice (data not shown). All of the Nrf2-deficient mice and none of the wild-type mice had died from progression of MeHg intoxication within 3 weeks of the first administration of MeHg (Figure 5B).

*SFN inhibits mercury accumulation* in vivo. If Nrf2 plays a role in the elimination of MeHg from the body, the accumulation of mercury in the organs should be increased by Nrf2 deficiency and decreased by Nrf2 activation. As expected, oral administration of MeHg (1 mg/kg) resulted in significantly greater accumulation of mercury in the cerebrum, cerebellum, and liver of Nrf2-deficient mice than in those of wild-type mice (Figure 5C–E). To examine the protective effect of ITC-mediated Nrf2 activation on MeHg accumulation *in vivo*, we administered SFN (5 mg/kg) to wild-type and Nrf2-deficient mice by intraperitoneal injection as previously described (Zhao et al. 2007). Injection of SFN before MeHg administration resulted in significant suppression of the accumulation of mercury in the cerebrum, cerebellum, and liver of wild-type mice (Figure 5C–E). In Nrf2-deficient mice, however, SFN did not affect the accumulation of mercury in the cerebrum and cerebellum and actually increased the accumulation of mercury in the liver (Figure 5C–E). Interestingly, pretreatment with SFN (20 mg/kg) significantly reduced not only acute MeHg intoxication as determined by hind-limb flaccidity but also mortality after oral administration of MeHg (50 mg/kg) to wild-type mice (Table 1).

**Table 1:** Hind-limb flaccidity and mortality of wild-type mice given MeHg.

Treatment		Hind-limb flaccidity		Mortality
SFN (20 mg/kg)		0/15		0/15
MeHg (50 mg/kg)		14/25		17/25
SFN pretreatment + MeHg		5/25**		10/25*
Wild-type mice received a single injection of SFN 16 hr before a single administration of MeHg. Experiments were carried out 2 days (hind-limb flaccidity) and 3 days (mortality) after injection of MeHg into mice. *p < 0.05 and **p < 0.01 compared with MeHg (50 mg/kg) alone.				

## Discussion

Our findings indicate that both 6-HITC and SFN activate Nrf2, resulting in up-regulation of GCL and the GSTs and MRPs responsible for conjugation of MeHg with GSH and excretion of the MeHg–GSH adduct into the extracellular space. Increased Nrf2 activation is associated with a reduction in cellular and organ levels of mercury and substantial suppression of MeHg-induced cytotoxicity and intoxication in primary mouse hepatocytes and in mice. Previous findings (Toyama et al. 2007) suggest that Nrf2 is a critical transcription factor in the reduction of MeHg-induced cytotoxicity and the excretion of MeHg into the extracellular space, because Nrf2 deletion significantly enhances MeHg accumulation and cytotoxicity in primary mouse hepatocytes. Our findings support this notion.

A common characteristic of MeHg-resistant cell lines is the reduced accumulation of MeHg compared with that in nonresistant parent cells (Miura and Clarkson 1993), indicating that protection against MeHg intoxication is, at least in part, associated with decreased influx and/or increased efflux of MeHg. Several researchers have reported that the GSH transport system is closely associated with MeHg efflux (Ballatori and Clarkson 1983, 1985; Fujiyama et al. 1994). Kaur et al. (2006) reported that diethylmaleate, used to deplete GSH levels, increased MeHg accumulation and enhanced MeHg-induced oxidative stress in primary cell cultures of neurons and astrocytes. Also, the GSH transport inhibitor phenol-3,6-dibromophthalein inhibits MeHg efflux from PC12/TM cells (a MeHg-resistant rat pheochromocytoma cell line), thereby increasing MeHg accumulation (Miura and Clarkson 1993). Consistent with these results, pretreatment with BSO (a GCL inhibitor), EA (a GST inhibitor), or MK-571 (an MRP antagonist) before MeHg exposure increased mercury accumulation and cytotoxicity in primary mouse hepatocytes (Figure 1). A reduction of steady-state levels of MeHg in cells is associated with diminished chemical modification of cellular proteins, confirming the importance of formation of the MeHg–GSH adduct, and its excretion into the extracellular space, in the detoxification of MeHg.

Even if cellular proteins are covalently modified by MeHg, MeHg–protein adducts are reversibly exchanged by GSH (Dallas 1978). This suggests that GSH protects against MeHg toxicity by increasing MeHg efflux and subsequently decreasing the levels of proteins modified by MeHg in the cell. Winroth et al. (1981) reported that the MeHg–protein adduct is a major form of MeHg in brain of monkeys administered MeHg. When GSH concentrations are low, the excess accumulation of MeHg–protein adducts may be the trigger for cell death. Consistent with this, Nrf2 deletion enhanced MeHg–protein adduct formation during exposure of primary mouse hepatocytes to MeHg, as evaluated by the biotin-maleimide labeling assay (Toyama T, unpublished data).

As shown in Figure 3, 6-HITC activated Nrf2 and up-regulated downstream proteins associated with the detoxification and excretion of MeHg, such as GCLM, GCLC, GSTA1, MRP1, and MRP2, in primary mouse hepatocytes. Similar results have been obtained using SFN (Shinkai et al. 2006). The mechanisms of action of these ITCs are thought to involve reversible modification of the cysteine residues of Keap1 by the carbon in the –N=C=S motif (Figure 2); this leads to the stabilization of Nrf2 and the subsequent activation of ARE in the promoter region (Nakamura and Miyoshi 2010). In the present study, both 6-HITC and SFN suppressed mercury accumulation and cytotoxicity in primary mouse hepatocytes after exposure to MeHg (Figure 4). In particular, SFN reduced mercury accumulation in the cerebrum, cerebellum, and liver *in vivo* (Figure 5). Although SFN has been reported to have an Nrf2-independent mechanism of chemoprevention (Myzak and Dashwood 2006), all of the protective effects of the ITCs in this study were abolished by Nrf2 deletion, suggesting that the protective effects of ITCs on MeHg accumulation and cytotoxicity are indeed Nrf2 dependent. In Nrf2-deficient primary mouse hepatocytes and liver, ITCs actually enhanced mercury accumulation after exposure to MeHg. A possible explanation for this observation is that ITCs may act as competitive inhibitors of MeHg efflux under low-GSH conditions, because ITCs themselves are also thought to be conjugated by GSH and excreted via MRPs (Nakamura and Miyoshi 2010).

Selenium and the omega-3 fatty acids, which are essential nutrients, have been shown to confer protection against MeHg toxicity (Kaur et al. 2007, 2008; Nishikido et al. 1987; Rice 2008; Strain et al. 2008). However, selenium has no ability to reduce MeHg accumulation in the body (Newland et al. 2006; Urano et al. 1997). Docosahexaenoic acid, an omega-3 fatty acid, has also been shown to decrease MeHg accumulation in several cell lines; however, the precise mechanism of this effect is not known (Kaur et al. 2007, 2008). In the present study, we demonstrated that ITCs such as 6-HITC and SFN are potentially useful chemopreventive agents for MeHg accumulation and toxicity and that they exert their action through an Nrf2-dependent mechanism. This finding may provide helpful information for risk management strategies to reduce the potential health risk of MeHg exposure.

## Conclusion

The present study indicates that ITCs are effective agents for the reduction of MeHg accumulation via an Nrf2-dependent mechanism *in vitro* and *in vivo*.
